# Bioactive Components and Functional Applications of Sea Buckthorn: A Review

**DOI:** 10.1002/fsn3.72289

**Published:** 2026-08-24

**Authors:** Xiaogang Guo, Laifeng Zhuo, Yanru Feng, Menghui Lin, Thanh Ninh Le, Minjie Zhao, Haiying Cai

**Affiliations:** ^1^ School of Biological and Chemical Engineering Zhejiang University of Science & Technology Hangzhou China; ^2^ Wushi Yuanmai Agriculture Forestry and Animal Husbandry Industry Development Co., Ltd Aksu China; ^3^ College of Biosystems Engineering and Food Science Zhejiang University Hangzhou China; ^4^ Institute of Biotechnology and Food Technology Thai Nguyen University of Agriculture and Forestry Thai Nguyen Vietnam

**Keywords:** bioactive components, food processing, mechanism research, sea buckthorn

## Abstract

Sea buckthorn ( *Hippophae rhamnoides* L.) is a medicinal and edible plant rich in polyphenols, vitamins, fatty acids, carotenoids, and polysaccharides. These constituents are associated with antioxidant, anti‐inflammatory, hepatoprotective, gut microbiota‐modulating, antibacterial, and other health‐related activities, supporting its potential use in functional foods, nutraceuticals, pharmaceuticals, and cosmetics. However, its composition and bioactivity vary with plant part, extract type, and processing method, which complicates comparison across studies. This review evaluates sea buckthorn as a food‐derived functional resource by integrating evidence on its bioactive compounds, biological mechanisms, processing technologies, safety considerations, and food applications. Unlike reviews mainly summarizing phytochemical profiles or individual bioactivities, this review adopts a component–matrix–function perspective, emphasizing plant‐part specificity, extract‐dependent compositional variation, and translational relevance. Current evidence suggests promising application potential, although standardized preparations, quality markers, and clinically relevant studies are still needed to support evidence‐based utilization.

## Graphical abstract

Sea buckthorn ( *Hippophae rhamnoides* L.) is a multifunctional food resource whose bioactive composition and functional potential depend strongly on plant part, extraction method, and processing matrix. Fruits and juices are rich in vitamin C, organic acids, carotenoids, phenolic acids, and flavonol glycosides; leaves provide abundant flavonoids, phenolic acids, and polysaccharides; seed and pulp oils contain distinct profiles of unsaturated fatty acids, phytosterols, tocopherols, and carotenoids; and pomace represents a valuable source of dietary fiber, polysaccharides, and residual polyphenols. Processing strategies, including hydroalcoholic extraction, supercritical CO_2_ extraction, fermentation, and nonthermal technologies such as PEF–HPP, can selectively enrich bioactive fractions or improve product quality. Current evidence supports antioxidant, anti‐inflammatory, hepatoprotective, metabolic, gut microbiota‐modulating, intestinal barrier‐protective, and antibacterial potential, providing a scientific basis for applications in juices, oils, fermented foods, fruit wines, by‐product‐derived functional ingredients, and nutraceutical products. However, standardized preparations, quality markers, and further well‐designed human studies are required to support evidence‐based functional claims and translational applications.

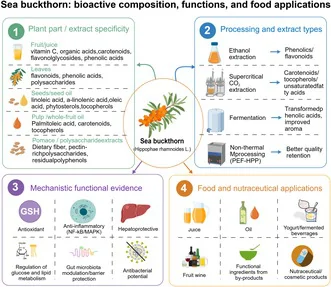

## 1 Introduction

Sea buckthorn ( *Hippophae rhamnoides* L.), a perennial deciduous shrub or small tree of the genus Hippophae (Elaeagnaceae), is widely distributed in the temperate and cold‐temperate zones of Eurasia. In China, it is predominantly found in high‐altitude, arid, and semiarid regions of the northwest, north, and southwest (Jain et al. 2022; Mei et al. 2023). As a typical ecological and economic plant, sea buckthorn possesses exceptional tolerance to drought, salinity, and infertile soils, rendering it a key species for soil and water conservation, windbreak and sand fixation, and large‐scale ecological restoration (Mei et al. 2023). Sea buckthorn has a long history of application in traditional medicine, including Tibetan and Mongolian medicine, for treating ailments such as cough, phlegm, tuberculosis, and gastrointestinal disorders. Its medicinal properties are also documented in the *Compendium of Materia Medica*, which records its warm nature, sour–sweet taste, and therapeutic effects of relieving cough and phlegm, promoting digestion, and resolving stagnation (Arimboor et al. 2008; Pundir et al. 2021).

Modern nutritional investigations have identified sea buckthorn as a nutrient‐dense plant source characterized by high concentrations of antioxidant vitamins, carotenoids, phenolics, and functional lipids. Its fruits are rich in vitamin C, vitamin E, and carotenoids, with vitamin C levels reported to exceed those of many commonly consumed fruits under comparable analytical conditions. Furthermore, its leaves are abundant in flavonoids, polysaccharides, and terpenes; however, reported total flavonoid levels and leaf‐to‐fruit ratios vary with cultivar, plant part, developmental stage, extraction solvent, and quantification method. Therefore, the statement that leaf flavonoid content may be approximately 2‐ to 3‐fold higher than that of fruits should be interpreted as study‐specific rather than universal. Sea buckthorn oil, derived from seeds, fruits, or whole fruits, is rich in unsaturated fatty acids (e.g., palmitoleic acid, linoleic acid, and α‐linolenic acid), endowing it with both nutritional and functional importance (Beveridge et al. 2002; Dabrowski et al. 2022; Gutzeit et al. 2008).

Modern scientific research has confirmed that the medicinal and health‐promoting effects of sea buckthorn are attributed to its diverse bioactive compounds. With advancements in natural product chemistry and pharmacological technologies, the identification of these bioactive components is constantly expanding, and research on their functional mechanisms is gradually advancing from in vitro studies to in vivo animal models and clinical trials (Dong et al. 2025; Mihal et al. 2023). Despite this progress, several knowledge gaps remain unresolved. First, the mechanisms of action of specific bioactive fractions, particularly flavonoid glycosides, polysaccharides, carotenoid‐rich oils, and palmitoleic‐acid‐rich lipid fractions, have not been consistently characterized across plant parts and extraction systems. Second, many studies report bioactivity without sufficient compositional standardization, making it difficult to associate marker compounds with biological endpoints. Third, evidence from in vitro assays and animal models has not yet been adequately integrated with clinical outcomes, safety data, and food‐processing feasibility. These unresolved issues limit the establishment of preparation‐specific quality markers, dose ranges, and application‐oriented claims for sea buckthorn‐derived foods and nutraceuticals. The utilization of sea buckthorn resources has expanded beyond traditional fresh fruit consumption to include a wide range of deeply processed products, such as sea buckthorn juice, sea buckthorn oil, dietary supplements, and cosmetics (Zhou et al. 2025; Zhu et al. 2025). These unresolved issues limit the establishment of preparation‐specific quality markers, dose ranges, and application‐oriented claims for sea buckthorn‐derived foods and nutraceuticals. Accordingly, a review that connects composition, matrix, mechanisms, processing, and translational relevance is needed to support the efficient utilization of sea buckthorn resources and the sustainable development of related industries.

This review aims to critically summarize the bioactive constituents of sea buckthorn and their functional evidence, with particular emphasis on plant‐part specificity, extract‐dependent composition, mechanistic pathways, food and nutraceutical applications, processing technologies, and safety considerations. The scope is delimited to evidence that links sea buckthorn‐derived components or matrices with compositional features, biological activity, processing performance, or translational relevance, rather than to a purely ethnobotanical or descriptive phytochemical inventory.

A structured review approach was used to guide the literature search. PubMed, Web of Science Core Collection, Scopus, Embase, and CNKI were searched from database inception to January 2026. The search strategy combined controlled vocabulary and free‐text terms related to sea buckthorn, its bioactive compounds, functions, and applications, including “sea buckthorn” OR “ *Hippophae rhamnoides* ” AND “bioactive compounds” OR polyphenols OR flavonoids OR carotenoids OR polysaccharides OR fatty acids OR oil OR extract AND antioxidant OR anti‐inflammatory OR hepatoprotective OR gut microbiota OR food application OR nutraceutical OR processing. Studies were considered eligible when they reported compositional characterization, biological activities, mechanistic evidence, safety data, or food/nutraceutical applications of sea buckthorn‐derived materials. Studies were excluded if they were unrelated to sea buckthorn, lacked primary data, or did not provide clear information on plant part, extract type, or outcome assessment.

**FIGURE 1:**
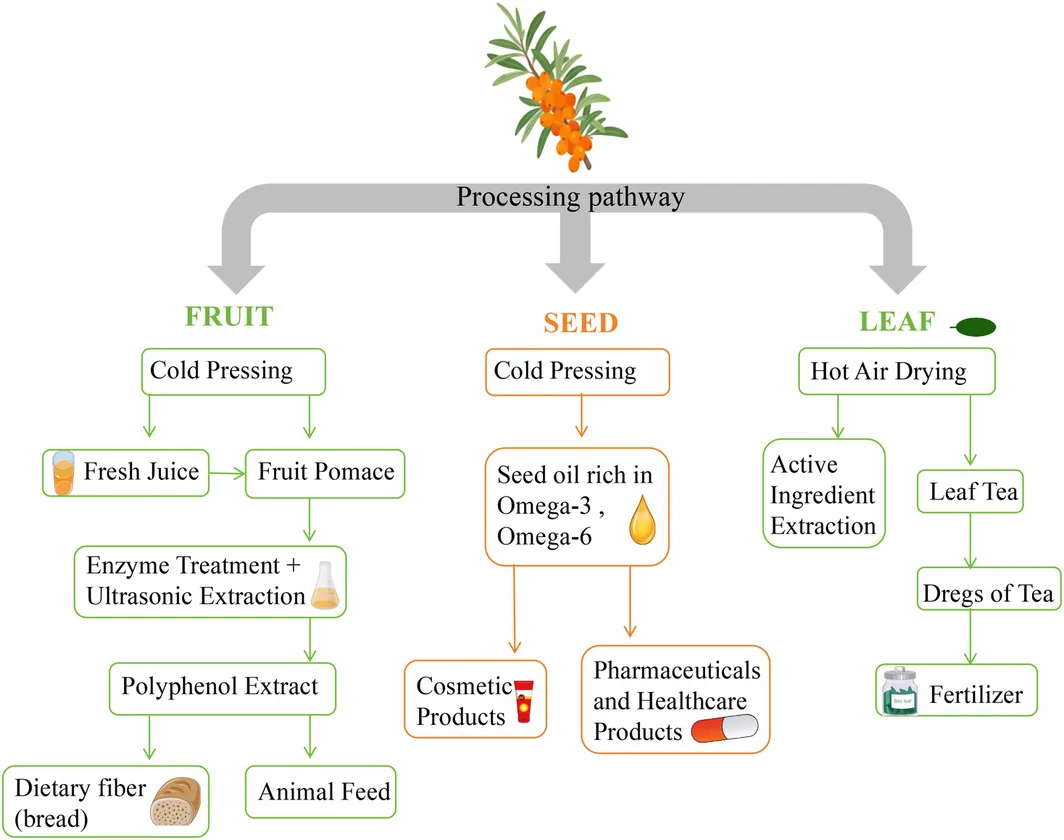
Flow chart of the whole component integrated processing of sea buckthorn raw materials.

The main contribution of this review is the use of a component–matrix–function analytical framework. Previous reviews, including those by Ma et al. (2022) and Dong et al. (2023), have provided valuable summaries of the phytochemical composition, nutritional properties, health‐promoting effects, and food applications of sea buckthorn. However, these reviews primarily emphasize general compositional profiles, traditional uses, or broad functional properties. In contrast, the present review organizes the evidence around (i) plant‐part specificity, including fruit pulp, peel, seeds, leaves, pomace, and oil; (ii) extract‐type dependence, including aqueous, hydroalcoholic, fermented, oil‐based, and supercritical CO_2_ extracts; (iii) quantitative ranges of representative bioactive compounds; and (iv) mechanistic evidence relevant to food, nutraceutical, processing, safety, and translational applications. By linking bioactive components, botanical or processing matrices, and functional outcomes, this review aims to clarify how compositional variability affects biological activity, product quality, translational relevance, and future standardization needs. The whole‐component integrated processing pathway that links sea buckthorn raw materials, bioactive fractions, and downstream applications is illustrated in Figure 1.

## 2 Analysis of Nutritional and Active Ingredients in Sea Buckthorn

The compositional profile of sea buckthorn is strongly influenced by the botanical matrix and extraction procedure; therefore, plant part and extract type should be specified when comparing studies. Fruits and fruit‐derived juices are typically characterized by vitamin C, organic acids, carotenoids, and phenolic acids; leaves tend to provide higher levels of flavonol glycosides; seeds and pulp oils differ in their fatty‐acid and phytosterol profiles; and pomace represents a relevant source of polysaccharides, dietary fiber, and residual polyphenols. These distinctions are important because aqueous, hydroalcoholic, fermented, oil‐based, and supercritical CO_2_ extracts enrich different groups of bioactive compounds and may therefore differ in functional properties (Liang, Deng, et al. 2022; Ma et al. 2022; Markkinen et al. 2021; Xu et al. 2024).

### 2.1 Polyphenolic Compounds

**FIGURE 2:**
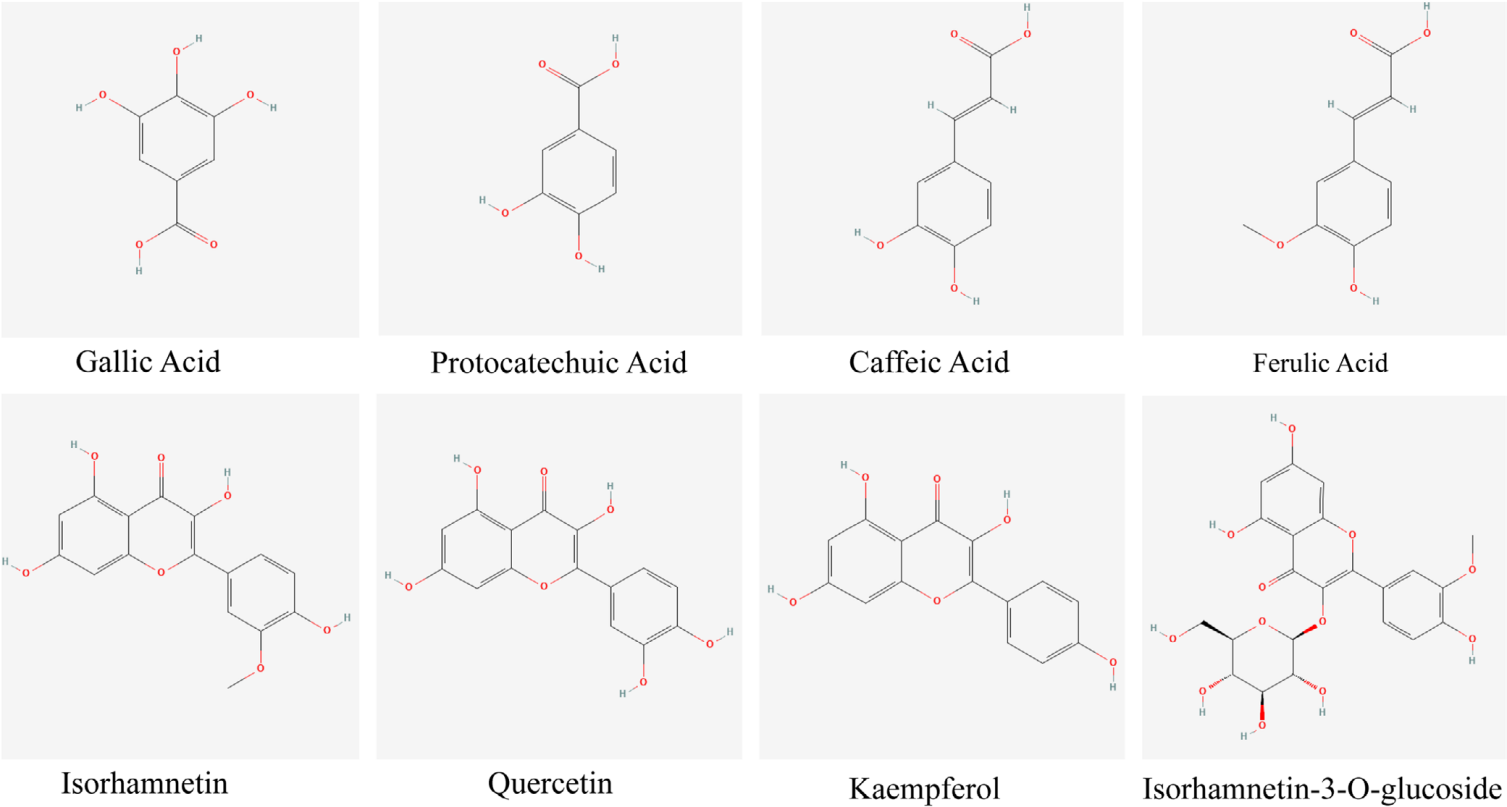
Description of the chemical structure of the main polyphenols of sea buckthorn. Data sourced from PubChem (https://pubchem.ncbi.nlm.nih.gov/).

Polyphenolic compounds are the most abundant and biologically active secondary metabolites in sea buckthorn (Raal et al. 2023), which are widely distributed in tissues (e.g., roots, stems, leaves, flowers, and fruits) and particularly enriched in the fruits. Over 100 polyphenolic components have been identified via techniques including UPLC‐MS/MS, HSCCC, and ^1^H‐NMR, among which phenolic acids and flavonoids are the core subclasses, accounting for more than 90% of the total polyphenolic content (Ma et al. 2022; Tkacz, Wojdyło, et al. 2020). Phenolic acids are dominated by gallic acid, protocatechuic acid, caffeic acid, and ferulic acid (Arimboor et al. 2008). Flavonoids primarily consist of glycoside derivatives of isorhamnetin, quercetin, and kaempferol, and in one recent fruit‐ and seed‐based dataset, isorhamnetin‐3‐O‐glucoside accounted for 66%–72% of the total flavonoid content; however, this proportion should be regarded as matrix‐ and sample‐specific rather than representative of all cultivars, geographic origins, maturity stages, or extraction systems. With characteristic components such as isorhamnetin‐3‐O‐glucoside accounting for 66%–72% of the total flavonoid content (Danielski and Shahidi 2024). Owing to their polyphenolic hydroxyl groups, sea buckthorn polyphenols exhibit potent antioxidant activity and possess pharmacological properties including anti‐inflammatory, lipid‐lowering, and immunomodulatory effects (Zhi et al. 2025). The bioactivity of sea buckthorn polyphenols is directly attributable to their phenolic hydroxyl groups, which provide the molecular basis for their potent antioxidant activity by donating hydrogen atoms and/or electrons to scavenge free radicals. For compositional interpretation, fruit‐ and juice‐derived extracts should be separated from leaf extracts: fruit materials mainly contribute phenolic acids and flavonol glycosides relevant to edible products, whereas leaves are generally richer in total flavonoids and are more suitable for tea, extract, and fermentation applications (Sytařová et al. 2020). Hydroalcoholic extraction favors the recovery of phenolics and flavonoids, whereas fermentation can modify phenolic‐acid profiles and improve the release or transformation of bound phenolics. The chemical structures of these representative phenolic acids and flavonoid monomers derived from sea buckthorn are illustrated in Figure 2.

### 2.2 Vitamins and Carotenoids

Vitamins and carotenoids are two core components in sea buckthorn that exhibit both nutritional and bioactive properties. They are widely distributed in the fruit flesh, peel, and leaves, with the highest concentrations in the fruits. Sea buckthorn is particularly rich in vitamin C, with a fruit content of 0.98–3.65 g kg^−1^—far exceeding that of common high‐vitamin C fruits and vegetables such as kiwifruits and lemons. Tocopherols and tocotrienols are concentrated mainly in lipid fractions rather than in juice or whole‐fruit aqueous matrices; therefore, oil‐based values are expressed consistently as mg/100 g oil. In Table 1, the value of 90–160 mg/100 g refers specifically to sea buckthorn oil, whereas values reported for whole fruit, freeze‐dried material, pulp oil, seed oil, or whole‐fruit oil should not be directly compared without conversion to the same matrix and unit (Sytařová et al. 2020; Xu et al. 2024). To date, multiple carotenoid components, including β‐carotene, lycopene, lutein, zeaxanthin, β‐cryptoxanthin, and γ‐carotene, have been identified in sea buckthorn berries. These pigments contribute substantially to the yellow, orange, and red coloration of sea buckthorn fruit flesh, although the dominant carotenoid may vary among cultivars and fruit colors (Andersson et al. 2009; Liang, Zhang, et al. 2022). From a plant‐part perspective, vitamin C is mainly associated with fruit pulp and juice, whereas carotenoids are concentrated in the pulp, peel, and oil fractions. Therefore, juice‐based products primarily deliver water‐soluble antioxidants and organic acids, while oil‐based products concentrate lipophilic carotenoids and tocopherols that contribute to color, oxidative stability, and lipid‐phase antioxidant activity.

### 2.3 Functional Lipids

Fatty acids are the core bioactive components in sea buckthorn oil, predominantly concentrated in the fruit flesh, seeds, and peel (Melnikova et al. 2025). However, sea buckthorn oil should not be treated as a single uniform lipid matrix. Seed oil is generally dominated by polyunsaturated fatty acids, especially linoleic acid (18:2n‐6) and α‐linolenic acid (18:3n‐3), whereas pulp or fruit oil contains higher proportions of monounsaturated fatty acids, particularly palmitoleic acid (16:1n‐7), together with palmitic acid (16:0) (Dulf 2012; Fatima et al. 2012; Yang and Kallio 2002). Reported unsaturated fatty acids account for 72.6%–83.4% of total fatty acids, but this range should be attributed to the specified oil fraction and cultivar rather than generalized to all sea buckthorn oils (Máté et al. 2022). In the cited data set, linoleic acid and α‐linolenic acid accounted for 17.0%–33.2% and 15.3%–24.9%, respectively, corresponding to an approximate linoleic acid/α‐linolenic acid ratio of 0.7–2.2. This indicates a relatively high contribution of n‐3 fatty acids; because dietary recommendations emphasize adequate intake of individual fatty acids rather than a fixed universal n‐6/n‐3 ratio, the nutritional relevance of this ratio should be interpreted within the overall diet (Máté et al. 2022). The frequently cited palmitoleic acid range of 32%–53% refers specifically to pulp oil, where palmitoleic acid can be the predominant fatty acid, and should not be applied to seed oil or unspecified whole‐fruit oil (Arimboor et al. 2008; Yang and Kallio 2002). As a naturally occurring ω‐7 fatty acid synthesized from palmitic acid via stearoyl‐CoA desaturase‐1 (SCD‐1), palmitoleic acid has been described as a lipokine involved in metabolic signaling; nevertheless, its biological significance should be discussed in relation to the relevant oil fraction and dose (Bermúdez et al. 2022; Zhang et al. 2024).

### 2.4 Phytosterols

Sea buckthorn phytosterols are lipophilic bioactive constituents that are chemically distinct from acyl lipids and are mainly distributed in lipid‐rich fractions of the seeds, pulp, and whole fruit. Recent studies have shown that sea buckthorn seed oil and pulp oil contain abundant phytosterols, with β‐sitosterol as the dominant sterol, while campesterol, stigmasterol/stanol derivatives, avenasterols, and cycloartanol‐type sterols are also detected. In particular, seed oil and pulp oil differ not only in fatty‐acid composition but also in their minor lipid profiles; therefore, phytosterol composition can serve as a useful nutritional attribute and a complementary quality marker for differentiating sea buckthorn oil fractions. Functionally, β‐sitosterol, campesterol, and stigmasterol are generally associated with cholesterol‐lowering effects because plant sterols can reduce intestinal cholesterol absorption by competing with cholesterol during micellar incorporation, although this effect depends on dose, food matrix, oil fraction, and co‐occurring lipids (Li et al. 2022; Wang, Zhao, et al. 2022; Wang, Zhou, and Jiang 2022).

### 2.5 Polysaccharides

Sea buckthorn polysaccharides consist of core backbones (e.g., pectin, galactan, and arabinan), which are frequently linked to branched residues (e.g., xylose and rhamnose) via glycosidic bonds (Shen et al. 2021). Some further conjugate with phenolic acids and proteins to form glycoconjugates. These polysaccharides exhibit good water solubility and water‐swelling capacity, with their bioactivity closely associated with molecular weight, uronic acid content, and branched structure (Ling et al. 2024). Polysaccharides are commonly obtained from fruits, pomace, and other water‐extractable fractions. Their reported activities should be interpreted together with extraction temperature, precipitation conditions, molecular‐weight distribution, monosaccharide composition, and uronic acid content, as these features can affect solubility, viscosity, fermentability, and interactions with gut microbiota.

Extraction and purification conditions are major determinants of polysaccharide composition. Conventional hot‐water extraction followed by ethanol precipitation remains widely used for crude polysaccharide recovery, whereas enzymatic, ultrasound‐assisted, microwave‐assisted, and combined extraction methods can improve extraction yield by disrupting cell‐wall pectin, cellulose, and hemicellulose networks. Subsequent deproteinization, dialysis, ion‐exchange chromatography, and gel filtration chromatography are commonly required to separate fractions with different charge density and molecular size. Because extraction temperature, pH, enzyme specificity, ultrasonic power, solvent‐to‐solid ratio, and alcohol‐precipitation concentration may alter molecular weight and uronic acid content, polysaccharide data should be reported together with the complete extraction and purification protocol (Ling et al. 2024; Teng et al. 2024; Yang et al. 2024).

The biological activity of sea buckthorn polysaccharides is therefore better interpreted through structure–activity relationships than through total polysaccharide yield alone. Fractions with higher uronic acid content and appropriate molecular weight may show stronger water‐holding, viscosity, bile‐acid‐binding, and gut‐microbiota‐modulating properties, whereas branching degree and glycosidic linkage types can influence solubility, fermentability, receptor recognition, and antioxidant capacity. In preclinical studies, sea buckthorn polysaccharides have been associated with antioxidant and anti‐inflammatory effects, intestinal barrier protection, gut microbiota regulation, and hepatoprotective activity; however, these effects cannot be generalized without defining the plant part, extraction process, molecular‐weight distribution, monosaccharide profile, and dose (Lan et al. 2022; Li et al. 2024; Shen et al. 2021; Xie et al. 2023).

Accordingly, future polysaccharide studies should combine compositional assays with methylation analysis, FT‐IR, NMR, HPSEC‐MALLS, monosaccharide profiling, and standardized in vitro or in vivo activity models. Such integration would allow sea buckthorn polysaccharides to be compared across fruits, pomace, leaves, and processed products and would clarify whether observed activities arise from the polysaccharide backbone itself, associated proteins or phenolics, or microbiota‐derived metabolites.

### 2.6 Cross‐Subsection Synthesis: Matrix‐Level Interactions Among Bioactive Classes

The nutritional and functional properties of sea buckthorn arise from interactions among compound classes rather than from a single dominant constituent. In fruit and juice matrices, vitamin C, phenolic acids, and flavonol glycosides may jointly contribute to radical‐scavenging capacity, with vitamin C helping maintain redox cycling of phenolic antioxidants. In oil‐rich matrices, fatty acids provide the lipid phase required for the dissolution, micellarization, and potential bioaccessibility of carotenoids and tocopherols, while tocopherols and carotenoids also protect unsaturated fatty acids from oxidation. Polysaccharides and dietary fiber can further influence viscosity, release kinetics, fermentation by gut microbiota, and the intestinal transformation of coexisting phenolics and lipids. Therefore, the component–matrix–function relationship should be considered when comparing sea buckthorn juice, leaf extracts, pomace fractions, seed oil, pulp oil, whole‐fruit oil, and fermented products (Bayram and Decker 2023; Wang, Zhao, et al. 2022; Yan et al. 2024; Yuan et al. 2025).

## 3 Functional Study of Sea Buckthorn

The health benefits of sea buckthorn are attributed to the synergistic interactions among its active compounds, but the available evidence remains uneven across functional endpoints and should not be interpreted as uniformly validated efficacy in humans. In this section, in vitro assays are treated as mechanistic screening evidence, animal studies as preclinical evidence, and human trials as preliminary clinical evidence when available. However, the strength of evidence differs across functional endpoints. Antioxidant and anti‐inflammatory activities are supported largely by chemical assays, cell models, and rodent studies, whereas dose–response data, clinically validated biomarkers, and human efficacy evidence remain limited. Distinguishing between preclinical and clinical evidence is important for avoiding overinterpretation of experimental findings.

### 3.1 Antioxidant Function

The antioxidant activity of sea buckthorn is primarily ascribed to its abundant bioactive constituents, including polyphenols, flavonoids, vitamin C, vitamin E, and carotenoids. These compounds exert antioxidant effects via multiple mechanisms: scavenging free radicals, inhibiting lipid peroxidation, and upregulating the activity of endogenous antioxidant enzymes. In one comparative study using the extraction and assay conditions specified by Singh et al. (2024). The antioxidant capacity ranked in the order of fruit oil > leaves > fruits; however, this order is assay‐ and sample‐dependent and should not be generalized across cultivars, extraction solvents, concentrations, or antioxidant test systems.

For in vitro antioxidant evaluation, Kreps et al. assessed the antioxidant activity of sea buckthorn juice extracts using electron paramagnetic resonance (EPR) and ultraviolet–visible spectroscopy (UV–VIS). Their findings revealed that the 70% ethanol extract exhibited the highest antioxidant capacity (123 mmol Trolox kg^−1^), significantly surpassing that of the 50% acetone extract (89 mmol Trolox kg^−1^). This activity showed a strong positive correlation with total flavonoid content (*r* = 0.978). Further investigation demonstrated that the extract displayed higher ABTS radical scavenging activity (98.3%) than DPPH radical scavenging activity (86.7%), indicating that the hydroxyl groups of flavonoids may have a higher affinity for ABTS (Kreps et al. 2021).

The antioxidant capacity of sea buckthorn oil is closely associated with its vitamin E and carotenoid contents. Research has indicated that fruit oil exhibits a significantly lower peroxide value than seed oil, attributed to its higher carotenoid concentration (Gâtlan and Gutt 2021). Meanwhile, sea buckthorn anthocyanins (SBPs) possess remarkable antioxidant activity, largely due to their structural characteristics—such as the relatively rare monomeric forms and a high degree of hydroxylation (Rösch et al. 2004). These properties enable SBPs to act as direct free radical scavengers to mitigate oxidative stress, as well as effective chelators of iron and copper ions, thereby inhibiting their catalytic effects in oxidation reactions (Perron and Brumaghim 2009).

Corroborating these in vitro observations, preclinical in vivo studies have suggested sea buckthorn's antioxidant potential: Ting et al. demonstrated that sea buckthorn seed oil mitigates carbon tetrachloride‐induced oxidative stress in mice, exhibiting concentration‐dependent enhancements in DPPH radical scavenging activity, ferrous ion chelation capacity, reducing power, and inhibition of lipid peroxidation (Ting et al. 2011). Zhang et al. (2017) reported that sea buckthorn berry polysaccharides protect against carbon tetrachloride‐induced hepatotoxicity in mice, a protective effect attributed to the extract's combined antioxidant and anti‐inflammatory activities. Although these findings support sea buckthorn as a promising source of natural antioxidants, the current evidence still relies predominantly on chemical assays and chemically induced animal models, while comparable human intervention studies using standardized oxidative stress biomarkers remain limited (Kreps et al. 2021; Zhang et al. 2017). Because carbon tetrachloride injury is an artificial toxicant model, these data should be interpreted as mechanistic and preclinical evidence rather than direct evidence for human liver disease prevention or treatment. Human intervention data remain limited: in a small controlled trial, sea buckthorn juice supplementation for 8 weeks produced a moderate decrease in the susceptibility of LDL to oxidation, but did not significantly change total cholesterol, LDL‐C, platelet aggregation, or ICAM‐1 levels (Eccleston et al. 2002). Collectively, these findings underscore the potential of sea buckthorn as a potent source of natural antioxidants. This supports its application in functional foods and nutraceuticals for mitigating chemical‐induced liver damage, as well as its broader use in the cosmetic and pharmaceutical industries. Therefore, antioxidant‐related applications should be described as potential functional uses pending confirmation in well‐powered clinical trials with standardized preparations and validated oxidative stress endpoints.

### 3.2 Anti‐Inflammatory Function

The anti‐inflammatory properties of sea buckthorn are primarily attributed to the inhibition of inflammatory mediator release and the modulation of key signaling pathways (Yang et al. 2025). Most mechanistic evidence is derived from cell‐based inflammation models and animal experiments, and therefore indicates anti‐inflammatory potential rather than established clinical efficacy. This bioactivity is largely conferred by its constituent flavonoids, particularly isorhamnetin and quercetin, the mechanisms of action of which are a subject of intensive investigation.

Jiang et al. employed an LPS‐induced RAW264.7 macrophage model to demonstrate that sea buckthorn flavonoids inhibit the production of NO and PGE_2_, and downregulate the mRNA expression levels of *iNOS* and *COX‐2*. Western blot analysis further revealed that these flavonoids suppress the phosphorylation of p38 and SAPK/JNK MAPK pathways, thereby reducing IκB‐α degradation and preventing the nuclear translocation of NF‐κB p65. This signaling cascade ultimately results in the suppression of key inflammatory cytokines, including TNF‐α, IL‐1β, and IL‐6 (Jiang et al. 2017). In a related study, Fan et al. reported that fermentation of sea buckthorn juice with *Lactobacillus plantarum Lp10211* increased the levels of protocatechuic acid and p‐coumaric acid. These fermented components exhibited a pancreatic lipase inhibition rate of 95.42% and significantly reduced LPS‐induced IL‐6 levels in RAW264.7 cells (*p* < 0.001). Taken together, these results provide mechanistic support for an anti‐inflammatory effect, yet the evidence at this stage remains largely confined to macrophage‐based models and pathway readouts rather than human inflammatory endpoints (Baek et al. 2020). A double‐blind randomized trial in healthy volunteers found that sea buckthorn berries did not reduce the number or duration of common cold or digestive tract infections, although serum C‐reactive protein decreased modestly compared with placebo (Larmo et al. 2008). This indicates that available human anti‐inflammatory evidence is limited and endpoint‐specific. This study indicates that the anti‐inflammatory effect may be mediated via the modulation of the TLR4–MyD88 pathway and subsequent inhibition of NF‐κB activation (Fan et al. 2024).

**FIGURE 3:**
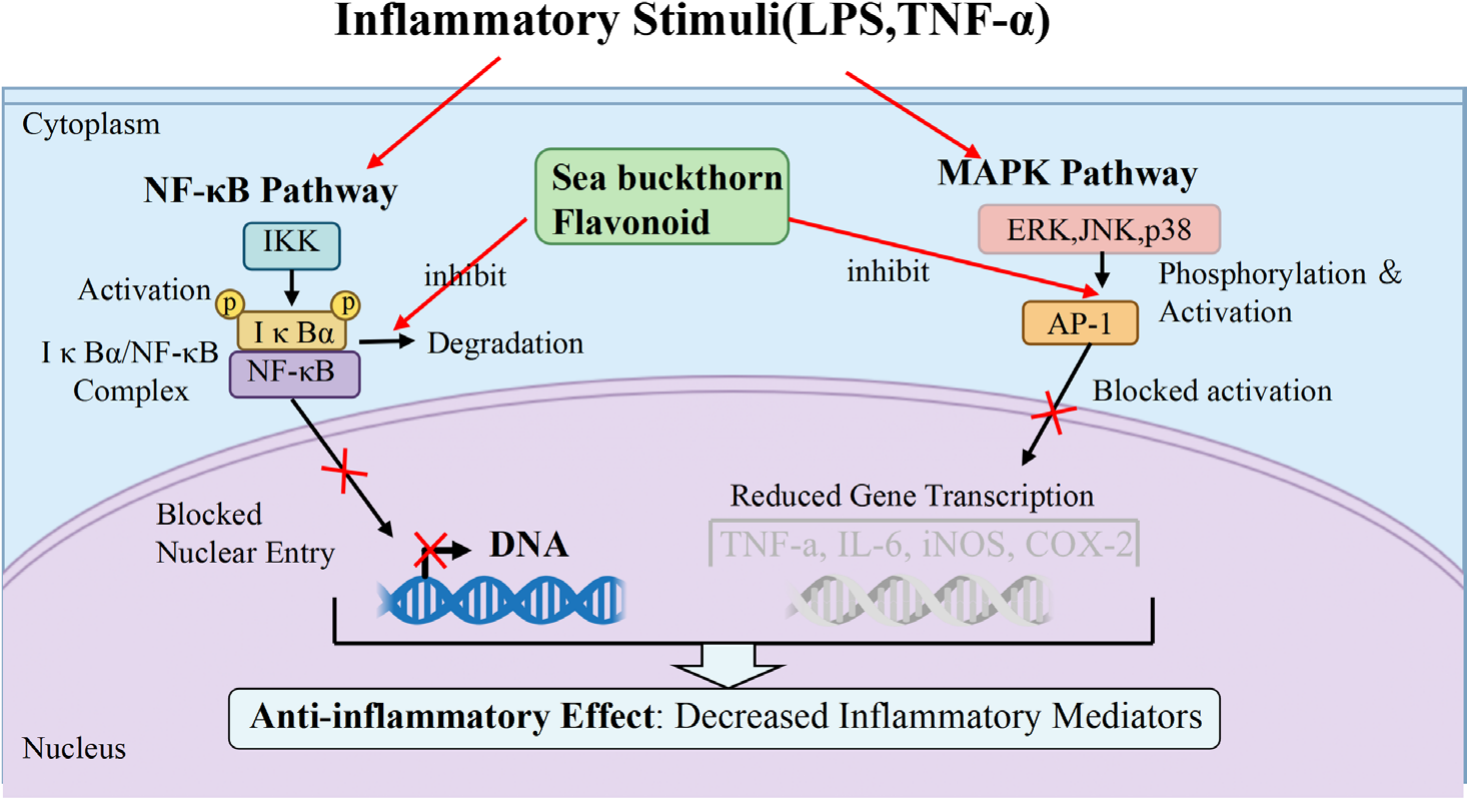
Anti‐inflammatory mechanism of sea buckthorn flavonoids via inhibition of NF‐κB and MAPK signaling pathways.

At the pathway level, available sea buckthorn‐specific evidence mainly indicates that sea buckthorn flavonoids (SBFs) suppress MAPK phosphorylation and NF‐κB p65 nuclear translocation in LPS‐stimulated macrophages, thereby reducing downstream mediators including TNF‐α, IL‐1β, IL‐6, COX‐2, and iNOS (Jiang et al. 2017). Figure 3 summarizes this proposed mechanism, but it should be interpreted as a cell‐model‐derived pathway rather than direct clinical proof of anti‐inflammatory efficacy (Chen et al. 2016; Zhou et al. 2019).

A mouse model of chronic obstructive pulmonary disease (COPD) was established through cigarette smoke (CS) exposure and intratracheal lipopolysaccharide (LPS) instillation. Isorhamnetin (Iso) was found to promote the ubiquitination of p62, leading to Keap1 degradation, thereby activating the Nrf2 pathway and upregulating protective factors, ultimately showing anti‐inflammatory activity in the lungs of CS‐exposed mice. It was further suggested that Iso alleviates inflammation in CS‐induced COPD mice primarily via the Nrf2/Keap1 pathway (Xu et al. 2022; Zhang et al. 2018). These findings remain preclinical and require validation in human respiratory or systemic inflammatory conditions before therapeutic conclusions can be drawn.

### 3.3 Regulation of Glucose and Lipid Metabolism

#### 3.3.1 Glucose Metabolism

Sea buckthorn has been reported to modulate glucose metabolism in cell and animal models by activating the PPARγ, PI3K/AKT, and Nrf2/HO‐1 pathways, as well as inhibiting α‐glucosidase activity. Sea buckthorn flavonoids and oil enhance glucose transporter 4 (GLUT4) translocation in muscle and adipose tissues via activation of the PPARγ and PI3K/AKT pathways, thereby increasing glucose uptake (Gao et al. 2017; Ollinger et al. 2022). Glucose‐6‐phosphatase (G6Pase) is a key enzyme regulating hepatic gluconeogenesis; sea buckthorn extract effectively inhibits G6Pase expression to modulate blood glucose metabolism (Cai et al. 2025). Additionally, sea buckthorn extract exhibits potent inhibitory activity against α‐amylase and promotes glucose consumption in HepG2 cells (Mu et al. 2021). Clinical evidence is still limited, although a randomized double‐blind crossover trial reported a modest decrease in fasting plasma glucose after 5 weeks of sea buckthorn fruit puree intake in individuals with impaired glucose regulation (Ren et al. 2021).

#### 3.3.2 Lipid Metabolism

A high‐fat diet severely impairs lipid metabolism, bile acid metabolism, and intestinal health. In rodent models, Sea buckthorn regulates lipid metabolism by promoting fat mobilization and β‐oxidation in high‐fat diet mice, while reducing hepatic lipid accumulation (Yuan et al. 2024). Peroxisome proliferator‐activated receptor α (PPARα) is a fatty acid‐regulated nuclear hormone receptor that controls lipid oxidation and the expression of fatty acid transport proteins. Sea buckthorn extract can activate PPARα, thereby inhibiting triacylglycerol (TG) synthesis and promoting fatty acid uptake (Mu et al. 2021). Because glucose regulation and lipid handling involve partly distinct pathways, future studies should report glycemic endpoints, lipid fractions, bile‐acid profiles, and gut‐microbiota changes separately instead of inferring broad metabolic efficacy from a single marker.

### 3.4 Liver Protection Function

Sea buckthorn has exerted hepatoprotective effects in models of alcoholic liver disease (ALD) and liver fibrosis, with the underlying mechanisms involving the regulation of lipid metabolism, inhibition of hepatic stellate cell activation, and modulation of gut microbiota. These findings highlight sea buckthorn as a promising candidate for the adjuvant treatment of liver diseases (Xu et al. 2014).

#### 3.4.1 Protection Against Alcoholic Liver Disease

The core pathological features of ALD include lipid accumulation, oxidative stress, and inflammatory responses, against which sea buckthorn has shown protective effects in preclinical studies by regulating fatty acid uptake, enhancing antioxidant capacity, and modulating gut microbiota (Ran et al. 2020). In a study using a mouse model of early alcoholic fatty liver disease (AFLD), sea buckthorn polysaccharides have been shown to restore the diversity, relative abundance, and community structure of intestinal mucosal microbiota. Notably, this study indicated that Astragalus polysaccharides may be more effective than sea buckthorn polysaccharides in the intervention of early AFLD; however, their combination appeared to attenuate the regulatory effect of Astragalus polysaccharides, leading to a more moderate modulation of gut microbiota in AFLD mice. The optimal dosage and ratio for such combination intervention require further investigation (Liu et al. 2022).

#### 3.4.2 Inhibition of Liver Fibrosis

The progression of liver fibrosis is primarily mediated by hepatic stellate cell (HSC) activation and excessive collagen deposition (Moreira 2007). Current sea buckthorn‐specific evidence for anti‐fibrotic activity remains limited and is derived mainly from preclinical studies and one small clinical report rather than from large randomized trials. Bioactive compounds in sea buckthorn may contribute to anti‐fibrotic effects through multiple mechanisms, including inhibiting HSC activation and inducing HSC apoptosis. Specifically, flavonoids and vitamin C in sea buckthorn efficiently scavenge free radicals (Wang, Wang, et al. 2022), alleviating oxidative stress in hepatocytes and thereby blocking upstream activation signals for HSC. Additionally, sea buckthorn extract exhibits significant anti‐inflammatory activity by suppressing the release of key pro‐inflammatory cytokines (e.g., TNF‐α and IL‐6), thereby interrupting the chronic inflammatory stimulation that sustains HSC activation. However, direct evidence that sea buckthorn constituents modulate HSC fate is still insufficient, and mechanistic extrapolation from non‐sea‐buckthorn extracts should be avoided. Furthermore, sea buckthorn polysaccharides may indirectly mitigate fibrosis by protecting hepatocyte membranes and promoting liver regeneration, thereby improving overall liver function (Gao 2003; Yoshioka et al. 2018; Zhang et al. 2018).

### 3.5 Regulating Gut Microbiota and Metabolites, Barrier Protection

Sea buckthorn has been reported to modulate the abundance and distribution of gut microbiota, thereby promoting intestinal peristalsis, enhancing intestinal barrier stability, and supporting overall health (Yuan et al. 2025). To determine whether the beneficial effects of sea buckthorn freeze‐dried powder are associated with gut microbiota modulation and the underlying mechanisms involving gut microbiota and their metabolites, an in vivo experiment was conducted by establishing two mouse models: a high‐fat diet (HFD) model and an HFD model supplemented with sea buckthorn freeze‐dried powder. Findings from this study revealed that sea buckthorn freeze‐dried powder modulates the abundance of specific gut bacterial taxa, as evidenced by a reduced Firmicutes/Bacteroidetes (F/B) ratio, increased relative abundance of Akkermansia, and decreased relative abundance of Desulfovibrio. Such alterations in gut microbiota composition can modulate the production of key metabolites, primarily short‐chain fatty acids (e.g., acetic acid, propionic acid, and butyric acid). Gut microbiota‐derived metabolites regulate host immune responses through multiple pathways and play a pivotal role in immunomodulation. Following intervention with sea buckthorn freeze‐dried powder, it was observed to reverse the HFD‐induced elevation of propionic acid and reduce butyric acid levels (Guo et al. 2020). This finding is consistent with the results reported by Lan et al. (2022). Numerous studies have confirmed that the protective and reparative effects of sea buckthorn on the intestine are primarily mediated by maintaining gut microbiota homeostasis, thereby preventing inflammation and modulating immune system responses (Ma et al. 2024). Overall, these findings support a microbiota‐mediated mechanism mainly in animal models and in vitro fermentation systems. Human clinical validation using metagenomic sequencing, standardized dietary controls, and defined sea buckthorn preparations is still lacking; therefore, claims regarding intestinal barrier protection and microbiota regulation should be framed as preclinical evidence.

### 3.6 Other Potential Effects: Antibacterial Activity and Human‐Reported Outcomes

#### 3.6.1 Antibacterial Activity

In vitro studies have verified that sea buckthorn extract exerts inhibitory effects against various pathogens, including Gram‐positive bacteria, Gram‐negative bacteria, and fungi (Stochmal et al. 2021). It also exhibits significant inhibitory activity against food spoilage bacteria, thereby extending food shelf life. The antibacterial activity of sea buckthorn essential oil was evaluated against five bacterial strains: *Bacillus subtilis* , *Bacillus cereus* , *Escherichia coli* , *Staphylococcus aureus* , and *Bacillus coagulans* . Results showed that the essential oil inhibited the growth of these five foodborne pathogens to varying degrees, and the oil derived from different sea buckthorn parts exhibited nearly identical inhibitory effects against *S. aureus* (Netreba et al. 2024). Because these findings are based on in vitro antimicrobial assays, they primarily support preservative or formulation potential and should not be interpreted as evidence of clinical anti‐infective efficacy.

#### 3.6.2 Skin, Cardiometabolic Markers, and Mucosal Outcomes

Sea buckthorn‐based functional health products exhibit multifaceted potential in promoting human health. Clinical trials have reported that 12‐week oral supplementation with sea buckthorn oil significantly enhances skin elasticity, delays skin aging, increases high‐density lipoprotein cholesterol (HDL‐C) levels, and elevates serum antioxidant enzyme activity. Participants also reported alleviation of mucosal dryness symptoms, such as dry eyes and vaginal dryness (Chan et al. 2024). Additional randomized trials of oil‐based products have reported attenuation of tear‐film osmolarity and dry‐eye symptoms, as well as improvement in vaginal‐health indicators in postmenopausal women (Larmo et al. 2010, 2014). However, these studies used different oil preparations, target populations, and endpoints, and their findings should not be generalized to juice, leaf extracts, polysaccharides, or fermented products. Collectively, these findings underscore the potential of sea buckthorn functional products in supporting cardiometabolic health, delaying skin aging, and relieving mucosal dryness. Larger, independently replicated trials are still needed to define effective dose ranges, active fractions, and clinically meaningful outcomes.

## 4 Applications and Processing Technologies of Sea Buckthorn in Food and Nutraceuticals

### 4.1 Sea Buckthorn Juice

Sea buckthorn juice is a functional beverage prepared from sea buckthorn fruits as the raw material. Its main nutritional components are presented in Table 1. Owing to its capacity to retain the nutritional value of raw materials and ease of consumption, it holds a prominent position among sea buckthorn deep‐processed products. It is rich in diverse bioactive constituents, including vitamin C, vitamin E, flavonoids, anthocyanins, phenolic acids, minerals, and phytosterols. Meanwhile, the organic acids contained in the juice (e.g., citric acid and malic acid) not only contribute to its characteristic sour and astringent flavor but also influence the sensory properties and stability of the product. Based on these components, sea buckthorn juice exhibits physiological functions such as scavenging free radicals, alleviating oxidative stress, and aiding in blood lipid regulation (Chen et al. 2023).

**TABLE 1:** Representative compounds in Sea Buckthorn and their primary sources of variation.

Nutrient category	Specific component	Content range of sea buckthorn fruits	Primary sources of variation	Reference(s)
Carbohydrates	Total soluble sugars (°Brix)	8.4–22.74 °Brix	Cultivar, maturity stage, geographic origin, fruit‐to‐juice ratio, dilution, enzymatic treatment, fermentation conditions, and storage	Beveridge et al. (2002); Markkinen et al. (2021); Wang et al. (2025)
	Glucose	10.5–29.8 mg/mL		Beveridge et al. (2002); Fan et al. (2024)
	Fructose	10.5–91.52 mg/mL		Beveridge et al. (2002); Liang, Deng, et al. (2022)
	Sucrose	1.67–15.40 mg/mL		Fan et al. (2024); Liang, Deng, et al. (2022)
	Dietary fiber	62–100 g/kg (dry weight)		Markkinen et al. (2021)
Organic Acids	Total titratable acidity (as malic acid)	1.97%–5.51%	Cultivar, geographic origin, maturity stage, juice dilution, thermal load, malolactic fermentation, analytical method, and storage conditions	Beveridge et al. (2002); Wang et al. (2025)
	Quinic acid	14.70–23.0 mg/mL		Beveridge et al. (2002); Wang et al. (2025)
	Malic acid	13.9–24.06 mg/mL		Beveridge et al. (2002); Wang et al. (2025)
	Citric acid	1.99–0.027 mg/mL (decreased after fermentation)		Beveridge et al. (2002); Feng et al. (2025)
	Oxalic acid	0.29–0.660 mg/mL		Beveridge et al. (2002); Feng et al. (2025); Tkacz, Chmielewska, et al. (2020)
	Tartaric acid	1.22–1.698 mg/mL		Beveridge et al. (2002); Tkacz, Chmielewska, et al. (2020)
Lipids	Total lipid content	2.38%–22.57% (oil content); seed oil: 70% (dry weight); pulp oil: 35% (dry weight)	Matrix type, plant part, seed/pulp/peel ratio, cultivar, drying basis, extraction method, solvent polarity, and whether whole fruit, seed, pulp, oil, or juice is analyzed	Markkinen et al. (2021); Xu et al. (2024)
	Palmitoleic acid	Pulp/fruit oil: 32%–53% of total fatty acids		Markkinen et al. (2021); Xu et al. (2024)
Proteins	Total protein	Seeds as high‐quality protein source; juice contains 18 types of amino acids	Plant part, seed/pulp contribution, cultivar, extraction method, juice clarification, protein precipitation during processing, and analytical basis	Markkinen et al. (2021)
	Essential amino acids	Leucine and lysine are prominently present		Markkinen et al. (2021)
Vitamins	Vitamin C (Ascorbic acid)	Mean: 173.3–174.2 mg/100 mL	Cultivar, harvest maturity, matrix type, oxygen exposure, pressing method, heat treatment, pH, light exposure, storage temperature, and storage time	Beveridge et al. (2002); Markkinen et al. (2021)
	Vitamin E (Tocopherols)	Oil matrix: 90–160 mg/100 g oil; whole fruit/dried material and different oil fractions are not directly interchangeable		Terechuk et al. (2019); Xu et al. (2024)
	β‐Carotene	860 mg/kg (dry weight in pulp); 16.48% increase after fermentation		Fan et al. (2024); Markkinen et al. (2021)
	Total carotenoids	160.5–329.4 mg/100 g (in oil); zeaxanthin and other carotenoids detected in juice		Fan et al. (2024); Terechuk et al. (2019)
Polyphenols	Total phenolics (TPC)	390.83–4269.21 mg GAE/L	Cultivar, plant part, pulp/peel contribution, solvent system, extraction temperature, fermentation, enzymatic treatment, oxidation during processing, and analytical method	Chan et al. (2024); Fan et al. (2024)
	Total flavonoids (TFC)	81.71–404.99 μg RE/mL; accounting for 76% of total flavonoids in sea buckthorn leaves		Markkinen et al. (2021); Xu et al. (2024)
	Specific polyphenol monomers	Isorhamnetin (392.22 mg/g), quercetin (124.03 mg/g), gallic acid, protocatechuic acid, etc.		He et al. (2023); Jiang et al. (2017); Xu et al. (2024)

Due to the short shelf life of freshly squeezed sea buckthorn juice, processing technologies are required to extend its shelf life. Traditional processing of sea buckthorn juice typically involves steps such as pressing, separation, and thermal sterilization (Linhares et al. 2020). However, thermal treatment tends to cause the loss of natural nutrients, color degradation, and aroma changes. Sea buckthorn‐specific evidence indicates that combined pulsed electric field and high‐pressure processing (PEF‐HPP) can increase juice yield, reduce enzymatic browning, and better retain characteristic volatiles and nutrients compared with thermal processing, although the magnitude of these effects depends on cultivar, pulp content, treatment intensity, and storage conditions (Zhang et al. 2024). In the same sea buckthorn juice study, PEF‐HPP did not markedly alter the six monitored organic acids, whereas thermal processing reduced malic and oxalic acids; therefore, conclusions regarding organic‐acid retention should be attributed to sea buckthorn‐specific data rather than extrapolated from other fruit systems. Thus, PEF‐HPP holds application potential for enhancing processing efficiency and comprehensive quality of sea buckthorn juice, but its industrial value should be assessed together with equipment cost, energy demand, throughput, and regulatory validation.

To mitigate the inherent sour and astringent taste of sea buckthorn fruits, blending with other fruit juices to produce mixed juice has emerged as a feasible product innovation direction (Feng et al. 2025). This approach can effectively harmonize flavors and improve consumer acceptability while retaining the original bioactive substances of sea buckthorn. Key aspects of sea buckthorn juice products include their main components, functional properties, processing technologies (as illustrated in the diagram), optimization strategies, bottlenecks, development directions, taste improvement, and malic acid reduction.

### 4.2 Sea Buckthorn Oil

Sea buckthorn oil is a lipid‐rich extract derived from the pulp, seeds, or whole fruits of sea buckthorn. It is not only abundant in oleic acid and linoleic acid but also contains a significantly higher content of palmitoleic acid than conventional vegetable oils. Linoleic acid, oleic acid, and γ‐linolenic acid are the major components of sea buckthorn oil, endowing it with core reparative, antioxidant, and anti‐inflammatory properties. It is widely applied in skincare products; orally administered, it contributes to cardiovascular health and enhances the overall antioxidant defense system (Solà Marsiñach and Cuenca 2019). The oil content of sea buckthorn seed oil is higher than that of sea buckthorn fruit pulp oil. Here, oil content refers to extractable lipid yield from the raw material rather than the lipid content of the oil itself.

Despite its rich nutritional value, the low oil yield of sea buckthorn oil severely constrains its industrial development. Currently, the main extraction processes for sea buckthorn oil include organic solvent extraction, pressing, enzymatic extraction, supercritical CO_2_ extraction, and ultrasonic‐assisted extraction. However, none of these methods are optimal for achieving high‐quality sea buckthorn oil products: the pressing method causes internal friction and temperature elevation, thereby reducing oil quality; the organic solvent extraction method has the issue of solvent residue (Akhter et al. 2023); supercritical CO_2_ extraction offers a high extraction rate and can efficiently retain bioactive constituents, but it is limited by low equipment capacity, high cost, and high energy consumption (Yin et al. 2005). Organic solvent extraction and supercritical CO_2_ extraction are often combined with ultrasonic or microwave‐assisted extraction to shorten extraction time and improve oil quality, but these combined methods have not been widely applied in industry (Isopencu et al. 2018). Thus, the production of high‐quality sea buckthorn oil requires the exploration of novel extraction processes or the optimization of existing methods.

### 4.3 Fermented Sea Buckthorn Beverages and Sea Buckthorn Yogurt

In recent years, fermentation is widely applied to various food raw materials to improve processing efficiency, quality, flavor, functionality, and storage safety (Cai et al. 2018). Sea buckthorn by‐products—such as pomace and leaves—have attracted increasing attention for their potential in the development of functional foods and beverages. Studies have shown that the incorporation of *Lactobacillus fermentum* into the traditional liquid fermentation process of sea buckthorn fruit vinegar not only increases the contents of total acids, acetic acid, and total flavonoids but also improves the sourness profile by significantly reducing irritating acids (e.g., citric acid and malic acid). This fermentation process further facilitates the formation of aromatic esters, thereby optimizing both the flavor and bioactive composition of the product (Feng et al. 2025). Additionally, fermenting sea buckthorn leaf extract using a symbiotic culture of bacteria and yeast (SCOBY) in a kombucha‐style fermentation significantly enhances its bioactive potential. This method markedly increases the contents of polyphenolic compounds (e.g., epigallocatechin gallate, rutin, and quercetin) while dynamically regulating mineral element contents, consequently improving the nutritional value and sensory quality of the final beverage simultaneously (Sanwal et al. 2025). Mechanistically, lactic acid fermentation can modify sea buckthorn matrices through malolactic conversion, glycosidase‐mediated release or transformation of bound phenolics, and microbial metabolism of sugars and organic acids. In sea buckthorn juice, *Lactobacillus plantarum* ‐mediated malolactic fermentation converts L‐malate into lactate and can increase pH, thereby reducing sharp acidity; in vinegar fermentation, the introduction of *Lactobacillus fermentum* has been linked with altered glycolysis and tricarboxylic‐acid‐cycle‐related metabolites, decreased malic/citric acid‐associated irritation, and increased ester formation. These reactions explain why fermentation may improve flavor and change phenolic profiles, but the outcome remains strain‐, substrate‐, and process‐dependent rather than universally beneficial (Feng et al. 2025; Markkinen et al. 2021).

The application of sea buckthorn in yogurt‐based products exhibits considerable innovative potential and diverse functional advantages. In probiotic yogurt production, sea buckthorn pulp acts as a natural additive that enhances nutritional value, improves microbial stability, and enriches sensory properties (K. Dong et al. 2023). For instance, probiotic yogurt prepared with sea buckthorn pulp using an ABT‐1 starter culture maintains excellent quality stability during refrigerated storage (Brodziak et al. 2021). Furthermore, sea buckthorn berries can be used to develop novel frozen yogurt with enhanced probiotic viability. When employed as an immobilization carrier for *Lactobacillus casei* ATCC 393, sea buckthorn helps maintain high probiotic cell viability even after 90 days of storage at −18°C, providing an effective approach to probiotic preservation in frozen yogurt products (Terpou et al. 2019). Additionally, combining sea buckthorn with other botanical ingredients such as elderberry and blackthorn plum puree in yogurt formulations has created new possibilities for flavor profiles, nutritional enhancement, and functional synergy. These developments fully illustrate the unique potential of sea buckthorn in nutritional fortification, probiotic protection, and product innovation within the yogurt industry (Najgebauer‐Lejko et al. 2021).

### 4.4 Sea Buckthorn Wine

Sea buckthorn wine is a distinctive fruit wine with prominent applications in food science and human health. Functionally, it is abundant in bioactive compounds (e.g., rutin and myricetin) and exhibits potent in vitro free radical scavenging activity. Animal studies have further demonstrated that administration of sea buckthorn wine alleviates chemically induced oxidative stress in animals and ameliorates high cholesterol diet‐induced hypercholesterolemia by increasing the HDL‐C/LDL‐C ratio, highlighting its potential in preventing oxidative damage and regulating blood lipid metabolism (Negi et al. 2013). To mitigate issues such as browning, astringent mouthfeel, and off‐odors associated with high polyphenol content, process optimization has been implemented via activated carbon of the Granucol series. This treatment effectively reduces polyphenol levels, enhances color properties, and attenuates yeast‐derived and fusel oil off‐flavors, thereby improving the sensory harmony and storage stability of the wine (Shkolnikova et al. 2019). However, activated carbon treatment should be evaluated as a trade‐off rather than only a sensory improvement: removal of astringency, browning precursors, and off‐odors may occur together with loss of bioactive polyphenols, which can reduce antioxidant potential and functional positioning of the final wine. Regarding flavor profiling, gas chromatography–mass spectrometry (GC–MS) and electronic nose analyses have revealed dynamic changes in volatile compounds during the production of sea buckthorn wine and its distillates. Fermentation and distillation have been shown to significantly increase the levels of key aroma components, particularly esters, providing a scientific basis for refining and stabilizing the sensory quality of the final product (Xia et al. 2022). Beyond its application as a traditional beverage, sea buckthorn wine exhibits promising potential for the development of functional foods and the advancement of modern fermentation technology.

### 4.5 Utilization of Sea Buckthorn By‐Products

Sea buckthorn by‐products have exhibited substantial application potential in food and functional ingredient development. When incorporated into bread formulations as a powder at an 8% addition level, sea buckthorn pomace significantly enhances the product's antioxidant activity, polyphenol content, and crude fiber content while maintaining satisfactory sensory acceptability. This strategy facilitates the high‐value utilization of processing by‐products in staple foods (Stanciu et al. 2023). Furthermore, the residues from sea buckthorn juice processing are rich in bioactive components such as polyphenols, flavonoids, and minerals. Ultrasonic‐assisted extraction has proven effective in concentrating these compounds, yielding extracts with remarkable antioxidant capacity that serve as quality raw materials for functional foods and dietary supplements (Luntraru et al. 2022). Additionally, dietary fiber—particularly soluble fiber—extracted from sea buckthorn pomace through optimized ultrasound‐enzymatic methods exhibits notable hypoglycemic activity. It demonstrates strong glucose adsorption capacity and inhibits α‐amylase and α‐glucosidase enzymes, showing promise for developing functional foods aimed at blood glucose regulation (Xiao et al. 2024). These diversified applications not only promote the resource utilization and recycling of sea buckthorn by‐products but also provide substantial support for innovation and health‐oriented development in the food industry. From a circular‐economy perspective, pomace, seeds, peels, and leaves should be treated as secondary raw materials rather than waste streams. Their valorization can reduce disposal pressure, improve material efficiency in juice and oil production chains, and generate added value ingredients such as dietary fiber, antioxidant extracts, seed proteins, and fermentation substrates. Future industrial assessment should therefore combine nutritional functionality with life cycle indicators, solvent/energy input, by‐product logistics, and economic feasibility.

### 4.6 Processing Technology of Sea Buckthorn

The extraction process critically affects the retention of bioactive constituents in sea buckthorn. 70% ethanol has been demonstrated to yield a significantly higher polyphenol extraction rate than 50% acetone, while minimizing pigment interference. Meanwhile, supercritical CO_2_ extraction (conducted at 28°C and 7 MPa) retains over 90% of carotenoids and vitamin E, exhibiting superior efficacy to conventional solvent extraction (Xu et al. 2024). A study compared the extraction efficiency of microwave‐assisted extraction (MAE), ultrasonic‐assisted extraction (UAE), Soxhlet extraction, and maceration for bioactive antioxidant constituents from different sea buckthorn parts. Results indicated that extracts obtained via MAE exhibited significantly higher antioxidant activity than those from UAE or maceration, and slightly outperformed those from Soxhlet extraction. These findings further confirm that microwave treatment efficiently releases bioactive substances from sea buckthorn (Sharma et al. 2008). Additionally, combined enzymatic hydrolysis has been shown to effectively disrupt the cellulose and pectin structures in sea buckthorn cell walls, increasing the soluble constituent content in the extract by 24%–80% compared with enzyme‐untreated samples (Ling et al. 2024). Therefore, future reports should specify the raw material, solvent polarity, extraction parameters, yield, and marker‐compound retention so that compositional data can be linked reliably to functional outcomes and processing suitability. The core advantages, limitations, and applicable raw materials of mainstream sea buckthorn processing technologies are systematically summarized in Table 2 for intuitive comparison.

**TABLE 2:** Critical comparison of processing technology categories, advantages, and limitations.

Processing technology category	Typical application products	Core advantages	Limitations/key considerations	Suitable sea buckthorn characteristics
Juicing Technology (Cold/Hot Pressing)	Freshly Squeezed Sea Buckthorn Juice, Mixed Fruit Juice	Simple operation, retains water‐soluble components (Vitamin C)	Juice yield is affected by fruit texture; heat treatment may cause vitamin C loss, browning, and aroma deterioration	High‐sugar, high‐juice yield varieties
Extraction Technology	Sea Buckthorn Oil, Polyphenol/Flavonoid Extracts	Enriches liposoluble/active components	Extraction efficiency and composition depend on plant part, solvent, and process parameters; quality control is required	High‐oil (Central Asian Subspecies), High‐polyphenol
Solvent Extraction	Industrial‐Grade Extracts (Low Cost)	Low cost, rapid large‐scale production	Solvent residues, recovery, and regulatory limits must be controlled; safe solvents are preferred for food applications	By‐products with large raw material volume (Fruit Pomace, Leaves)
Supercritical CO_2_ Extraction	High‐End Sea Buckthorn Oil (No Residues)	Environmentally friendly, high retention rate of active components	High equipment cost and energy demand; limited throughput; economic feasibility should be evaluated	Raw materials for high‐end health products
Ultrasonic/Microwave‐Assisted Extraction	Polyphenol, Polysaccharide Extracts	High efficiency, shortens extraction time	Scale‐up may involve uneven energy distribution and degradation of heat‐sensitive compounds	Laboratory/small‐to‐medium‐scale production
Fermentation Technology	Sea Buckthorn Yogurt, Fruit Wine, Fruit Vinegar	Improves taste (reduces acidity), enhances functionality	Outcomes depend on strain and process conditions; off‐flavor, excessive acidification, and microbial safety require control	High‐acid, high‐polyphenol varieties
Nonthermal Processing (PEF‐HPP)	Preservative Sea Buckthorn Juice	Reduces thermal damage, extends shelf life	High equipment cost; some enzymes may not be fully inactivated; shelf life and regulatory suitability require validation	Freshly squeezed juice, products requiring long‐term preservation
By‐Product Utilization Technology	Fruit Pomace Dietary Fiber, Leaf Tea, Feed	Resource recycling, reduces costs	Contaminants, compositional variation, and storage stability must be controlled; standardized processing is needed	By‐products after juicing/extraction (Fruit Pomace, Leaves, Seeds)

### 4.7 Safety Considerations for Food and Nutraceutical Applications

Given the increasing use of sea buckthorn in oils, beverages, supplements, and topical products, safety evaluation should be considered in a preparation‐specific manner rather than generalized across all sea buckthorn materials. The available toxicological evidence is encouraging, although it remains concentrated mainly on oil preparations. In rodents, seabuckthorn oil showed a maximum tolerated dose of > 20 mL/kg in an acute oral toxicity study and a no‐observed‐adverse‐effect level (NOAEL) of 10 mL/kg body weight in a 90‐day repeated‐dose study (Zhao et al. 2017). Under the tested experimental conditions, seabuckthorn berry oil did not show genotoxic or teratogenic signals (Wen et al. 2020).

Human evidence on dosage and tolerability is still limited to a small number of short‐term intervention studies, most of which used oil‐based products. In randomized trials, sea buckthorn oil was administered at 2 g/day for 3 months in individuals with dry eye and at 3 g/day for 3 months in postmenopausal women with vaginal atrophy, with overall acceptable short‐term tolerability (Larmo et al. 2010, 2014). A recent 12‐week randomized trial also indicated that short‐term oral sea buckthorn oil may be tolerated in studies assessing skin, ocular, and vaginal outcomes, although the tested formulation and study population differed from earlier trials (Chan et al. 2024). At the same time, safety findings should not be extrapolated across plant parts, extract types, doses, or target populations. Because isorhamnetin derivatives isolated from sea buckthorn have shown antiplatelet and anticoagulant activity in whole blood assays, concomitant use with antiplatelet or anticoagulant drugs warrants caution until clinically validated interaction data become available. Future studies should further clarify preparation‐specific dose ranges, long‐term tolerability, and safety in vulnerable populations (Stochmal et al. 2022).

## 5 Summary and Prospect

Although available evidence indicates broad functional potential for sea buckthorn, the research landscape remains heterogeneous, and several issues require further clarification. Taken together, the strongest evidence currently lies in matrix‐specific compositional characterization and in vitro or animal‐based mechanisms related to antioxidant activity, inflammation modulation, lipid metabolism, and gut microbiota regulation. In contrast, human evidence remains limited, heterogeneous, and concentrated mainly on oil‐based products, fruit puree, or juice interventions with relatively short durations and different endpoints. Therefore, sea buckthorn should be interpreted as a promising food‐derived functional resource rather than as a clinically validated intervention for disease prevention or treatment.

Over the past 3–5 years, the most important advances have shifted the field from descriptive phytochemical profiling toward plant‐part‐specific and extract‐specific interpretation. Recent studies have clarified differences among seed oil, pulp oil, whole‐fruit materials, leaf extracts, fermented juices, and pomace‐derived polysaccharides; they have also expanded attention to bioaccessibility, gut microbial transformation, fermentation‐mediated phenolic conversion, and by‐product valorization. However, many studies still use poorly standardized extracts, report only total compound classes, or infer functional efficacy from single assays, which limits cross‐study comparison and translation into food or nutraceutical claims.

Future studies would benefit from moving beyond descriptive compositional profiling toward standardized, mechanism‐based, and application‐oriented research. Clinical validation should be prioritized for four fractions with the clearest translational relevance: flavonoid‐rich fruit/leaf extracts, polysaccharide‐rich pomace or berry fractions, carotenoid‐ and tocopherol‐rich oil fractions, and palmitoleic‐acid‐rich pulp oil. Dose selection should be anchored to existing human studies where possible, such as sea buckthorn oil at approximately 2–3 g/day, fruit puree at 90 mL/day, or juice at 50 mL/day, while preclinical doses should be reported in mg/kg or dietary percentage and converted to human‐equivalent exposure before clinical design (Larmo et al. 2010, 2014; Ren et al. 2021; Chan et al. 2024). Promising delivery strategies include emulsion‐based oil systems, microencapsulation, protein‐polysaccharide carriers, and fermentation‐assisted release of bound phenolics, but these approaches should be evaluated using gastrointestinal digestion, Caco‐2 transport, pharmacokinetic, and microbiome‐based models rather than retention data alone.

Important priorities include establishing preparation‐specific quality markers; developing validated analytical methods to distinguish fruit‐, leaf‐, seed‐, pomace‐, oil‐, and fermentation‐derived products; integrating metabolomics, lipidomics, gut microbiome analysis, and pharmacokinetic approaches; and conducting randomized controlled trials with clearly defined dosage, intervention duration, safety monitoring, and biomarker‐based outcomes. A practical standardization framework should include authenticated botanical origin, cultivar and maturity information, plant part, extraction yield, solvent or processing parameters, marker compounds, contaminant limits, and batch‐to‐batch specifications. ISO‐style analytical validation, pharmacopoeial monograph principles, Good Agricultural and Collection Practices, and food‐grade GMP documentation could together provide a basis for comparing sea buckthorn products across studies and markets.

From an industrial perspective, further optimization of green extraction, nonthermal processing, encapsulation, controlled fermentation, and high‐value utilization of pomace and leaves may improve the stability, bioaccessibility, sensory quality, and sustainability of sea buckthorn‐based foods and nutraceuticals. In this context, a matrix‐specific and extract‐specific evaluation strategy will be essential for translating sea buckthorn from a broadly described botanical resource into standardized, evidence‐based functional food ingredients.

Safety and interaction issues also require more systematic treatment. Although acute, subchronic, genotoxicity, and teratogenicity studies of sea buckthorn oil have not identified major safety signals under the tested conditions, and short‐term human oil interventions have generally reported acceptable tolerability, these findings should not be extrapolated to all plant parts, concentrated extracts, vulnerable populations, or long‐term high‐dose use (Larmo et al. 2010, 2014; Wen et al. 2020; Zhao et al. 2017). Potential interactions with anticoagulant or antiplatelet drugs are particularly relevant because isorhamnetin derivatives from sea buckthorn have shown antiplatelet and anticoagulant activities in experimental systems; plausible but unconfirmed interactions with lipid‐lowering or antihypertensive agents should also be considered because sea buckthorn oils, phytosterols, and flavonoids may influence lipid absorption, vascular function, or drug‐metabolizing pathways. These possibilities remain insufficiently validated and should be tested through targeted ADME, platelet‐function, and clinical interaction studies.

## Author Contributions

**Thanh Ninh Le:** supervision. **Minjie Zhao:** supervision, investigation. **Haiying Cai:** validation, project administration, data curation, methodology, conceptualization, resources, writing – review and editing. **Menghui Lin:** data curation, writing – review and editing. **Laifeng Zhuo:** writing – review and editing, writing – original draft. **Xiaogang Guo:** software, writing – original draft, writing – review and editing, visualization. **Yanru Feng:** conceptualization, methodology.

## Funding

This work was supported by National Natural Science Foundation of China (32172214, 31972079). The funders had no role in the study design, data collection, data analysis, interpretation, and writing of the report.

## Ethics Statement

This article is a review of previously published literature and did not involve new experiments using human participants, animals, or clinical samples; therefore, ethical approval and informed consent were not required. The authors declare that they have no known competing financial interests or personal relationships that could have appeared to influence the work reported in this paper. All cited sources were used for scholarly synthesis and are acknowledged in the reference list.

## Conflicts of Interest

The authors declare no conflicts of interest.

## Data Availability

Data available on request from the authors.
